# Efficacy and safety of catheter ablation for atrial fibrillation in patients with heart failure with preserved ejection fraction: a systematic review and meta-analysis

**DOI:** 10.3389/fcvm.2024.1423147

**Published:** 2024-07-25

**Authors:** Xiaomei Chen, Xuge Zhang, Xiang Fang, Shenghong Feng

**Affiliations:** ^1^ Department of Cardiology, Dazhou Second People’s Hospital, Dazhou, China; ^2^ Department of Otorhinolaryngology Head and Neck Surgery, Dazhou Second People’s Hospital, Dazhou, China

**Keywords:** catheter ablation, atrial fibrillation, heart failure with preserved ejection fraction, efficacy, safety

## Abstract

**Background:**

Catheter ablation (CA) effectively treats atrial fibrillation (AF) in heart failure (HF) with reduced ejection fraction (HFrEF), improving clinical outcomes. However, its benefits for AF patients with heart failure with preserved ejection fraction (HFpEF) are still unclear.

**Methods:**

We systematically searched PubMed, Embase, Web of Science, the Cochrane Library, and Scopus for studies investigating outcomes of CA in AF patients with HFpEF. Efficacy indicators included freedom from AF and antiarrhythmic drugs (AAD) free AF elimination. Safety indicators comprised total complications, HF admission, all-cause admission, and all-cause mortality. Sixteen studies with 20,796 patients included in our research.

**Results:**

The comprehensive analysis demonstrated that, when comparing CA with medical therapy in HFpEF, no significant differences were observed in terms of HF admissions, all-cause admissions, and all-cause mortality [(OR: 0.42; 95% CI: 0.12–1.51, *P *= 0.19), (HR: 0.78; 95% CI: 0.48–1.27, *P *= 0.31), and (OR: 1.10; 95% CI: 0.83–1.44, *P *= 0.51)], while freedom from AF was significantly higher in CA (OR: 5.88; 95% CI: 2.99–11.54, *P* < 0.00001). Compared with HFrEF, CA in HFpEF showed similar rates of freedom from AF, AAD-free AF elimination, total complications, and all-cause admission were similar [(OR:0.91; 95% CI: 0.71,1.17, *P* =0.47), (OR: 0.97; 95% CI: 0.50–1.86, *P *= 0.93), (OR: 1.27; 95% CI: 0.47–3.41, *P* = 0.64), (OR: 1.11; 95% CI: 0.72, 1.73; *P *= 0.63)]. However, CA in HFpEF was associated with lower rates of HF admission and all-cause mortality [(OR: 0.35; 95% CI: 0.20, 0.60; *P *= 0.0002), (OR: 0.40; 95% CI: 0.18, 0.85; *P* = 0.02)]. Compared with patients without HF, CA in HFpEF patients exhibited lower rates of AAD-free AF elimination (OR: 0.48; 95% CI: 0.30, 0.75; *P* = 0.001). However, their rates of freedom from AF and total complications were similar [(OR: 0.70; 95% CI: 0.48, 1.02; *P* = 0.06), (OR: 0.60; 95% CI: 0.19, 1.90; *P* = 0.38)].

**Conclusion:**

This meta-analysis conducted provided a comprehensive evaluation of the efficacy and safety of CA in patients with AF and HFpEF. The results suggest that CA may represent a valuable treatment strategy for patients with AF and HFpEF.

**Systematic Review Registration:**

https://www.crd.york.ac.uk/PROSPERO/#recordDetails, identifier (CRD42024514169).

## 1 Introduction

Constituting at least 50% of prevalent instances, HFpEF represents a substantial share of global heart failure cases and is associated with an unfavorable prognosis (1, 2). Effectively addressing HFpEF requires a holistic strategy that prioritizes the screening and management of underlying causes and associated comorbidities in patients (3, 4). Due to the shared risk factors and common pathophysiological processes between AF and HF, AF frequently coexists in HFpEF patients. The prevalence of AF in HFpEF patients ranges from 40%–60%. Moreover, it is associated with heightened morbidity, thromboembolic susceptibility, hospitalization frequencies, and mortality rates (5–9). Recently, there has been growing interest in determining the optimal therapeutic approach for patients with AF and HFpEF.

CA is an effective and relatively safe therapeutic option for AF (10). It has increasingly become a successful and common strategy for managing symptomatic AF (11). HF may heighten the likelihood of complications related to CA. While researches have indicated that CA interventions can lead to a reduction in AF burden and confer benefits in terms of decreased rehospitalization and mortality rates among AF patients with HFrEF. However, there is scarce data on whether CA treatment for AF can offer clinical advantages for HFpEF patients (12, 13).

To assess the efficacy, a single-arm meta-analysis (14) aggregated multiple studies on CA utilization in patients with AF and HFpEF. The analysis indicated that CA can be beneficial in maintaining sinus rate. However, being a single-arm study, it is subject to certain limitations, including the absence of a control group and notable heterogeneity among the included studies. Up to date, there were few published studies and meta-analyses that related to the outcomes of CA in patients with AF and HFpEF, the results were inconsistent, and the available studies were inadequate to ascertain the superiority of catheter ablation in these patient cohorts. This meta-analysis aims to evaluate both the efficacy and safety of CA compared to medical therapy for patients with AF and HFpEF. Additionally, the study sought to assess the efficacy and safety of CA in patients with AF, encompassing both HFpEF and HFrEF, as well as individuals with HFpEF and without HF.

## 2 Methods

### 2.1 Literature search

In accordance with the PRISMA 2020 statement (15), our evidence-based analysis was conducted and prospectively registered in PROSPERO (CRD42024514169). The PRISMA 2020 checklist guided our methodology, ensuring transparency and rigor throughout the review process. Up to December 18, 2023, our systematic literature search comprehensively covered five databases, including PubMed, Embase, Scopus, Cochrane Library, and Web of Science.

The principal objective of this study was to systematically identify and review research studies examining the efficacy and safety of CA in the context of AF among patients with HFpEF. Only English-language publications were considered. The following terms were used to search the databases: “Atrial fibrillation”, “AF”, “Heart Failure”, “HF”, “heart failure with preserved ejection fraction”, “HFpEF”, “Catheter ablation” and “CA.” A detailed outline of the search strategy is provided in Supplementary Table S1.

Additionally, all the eligible articles were manually reviewed to ensure comprehensiveness. Two investigators conducted the search and assessment of included literature independently. Any discrepancies encountered during the literature search or assessment process were resolved through mutual consensus and discussion.

### 2.2 Identification of eligible studies

The criteria for eligible studies included: (1) randomized controlled, cohort, or case-control study design; (2) studies involving AF patients with HFpEF, HFrEF, and without HF; (3) studies examining CA and medical therapy(rhythm control using AAD) for AF patients with HFpEF, CA for AF patients with HFpEF and HFrEF (EF 40%–49% included in the HFrEF group), and CA for AF patients with HFpEF and without HF; (4) Studies that provided dependable information on outcomes (freedom from AF,AAD-free AF elimination, total complications, HF admission, all-cause admission, and all-cause mortality); (5) Studies had to be published in a peer-reviewed scientific journal.

We excluded reviews, letters, editorial comments, case reports, conference abstracts, unpublished articles, and articles not written in English.

### 2.3 Endpoints

The primary endpoint was efficacy–freedom from AF and AAD-free AF elimination. Freedom from AF was defined as absence of any symptomatic or asymptomatic atrial arrhythmia lasting more than 30 s after completing the blanking period (3 months) after CA or medical treatment. AAD-free AF elimination refered to achieving the elimination of AF without the use of AAD by the end of follow-up.

The secondary endpoint was safety outcomes. Safety outcomes included total complications after CA, HF admission, all-cause admission, and all-cause mortality. Total complications varied among studies, encompassing cardiac perforation/tamponade, access site/vascular complications, pericarditis, esophageal atrial fistula, pulmonary vein stenosis, phrenic nerve injury, acute heart failure, stroke/transient ischemic attack, air embolism, and prolonged hospitalization.

### 2.4 Data extraction

Data extraction was independently conducted by Xiaomei Chen and Xuge Zhang. Discrepancies were settled by the third investigator, Shenghong Feng, to achieve a conclusive resolution. The data extracted from the included studies encompassed the first author's name, publication year, country, study period, study design, sample size, age, body mass index (BMI), comorbidities, AAD usage, AF type, duration of AF before intervention, follow-up duration, left atrium (LA) volume, E/E', left ventricular end-diastolic dimension (LVEDd), procedure time, the primary endpoint and the secondary endpoint. When continuous variables were presented as median with range or interquartile range in the study, we derived the mean ± standard deviation using a validated mathematical approach (16, 17). In instances where data were either missing or unreported, we reached out to the corresponding authors to acquire complete data sets, if feasible.

### 2.5 Quality assessment

Two reviewers, Xiaomei Chen and Xuge Zhang, independently conducted the quality assessment of included studies. Randomized controlled trials underwent evaluation using the Cochrane risk of bias tool (18). Meanwhile, observational studies were assessed using the Newcastle-Ottawa Scale (NOS) (19), which employs 3 domains of selection, comparability and outcome/exposure.

### 2.6 Statistical analysis

The evidence synthesis utilized Review Manager version 5.4 (Cochrane Collaboration, Oxford, UK). Continuous variables were compared using weighted mean differences (WMD), while dichotomous variables were analyzed with odds ratios (OR) or hazard ratio (HR), both accompanied by 95% confidence intervals (CIs). Heterogeneity was assessed using the chi-squared (*χ*^2^) test (Cochran's Q) and inconsistency index (*I*^2^), with significant heterogeneity defined as *χ*^2^ *P*-value < 0.05 or *I*^2^ > 50%. Considering the heterogeneity among different studies, all data synthesis was performed using a random-effects model. Subgroup or sensitivity analyses were performed to evaluate the influence of individual studies on outcomes exhibiting significant heterogeneity. To examine publication bias, funnel plots were visually assessed using Review Manager version 5.4, while Egger's regression tests were employed with Stata version 17.0 (StataMP-64) for outcomes involving three or more studies. A significance threshold of *P *< 0.05 denoted the presence of statistically significant publication bias.

## 3 Results

### 3.1 Literature search and study characteristics

**Figure 1:**
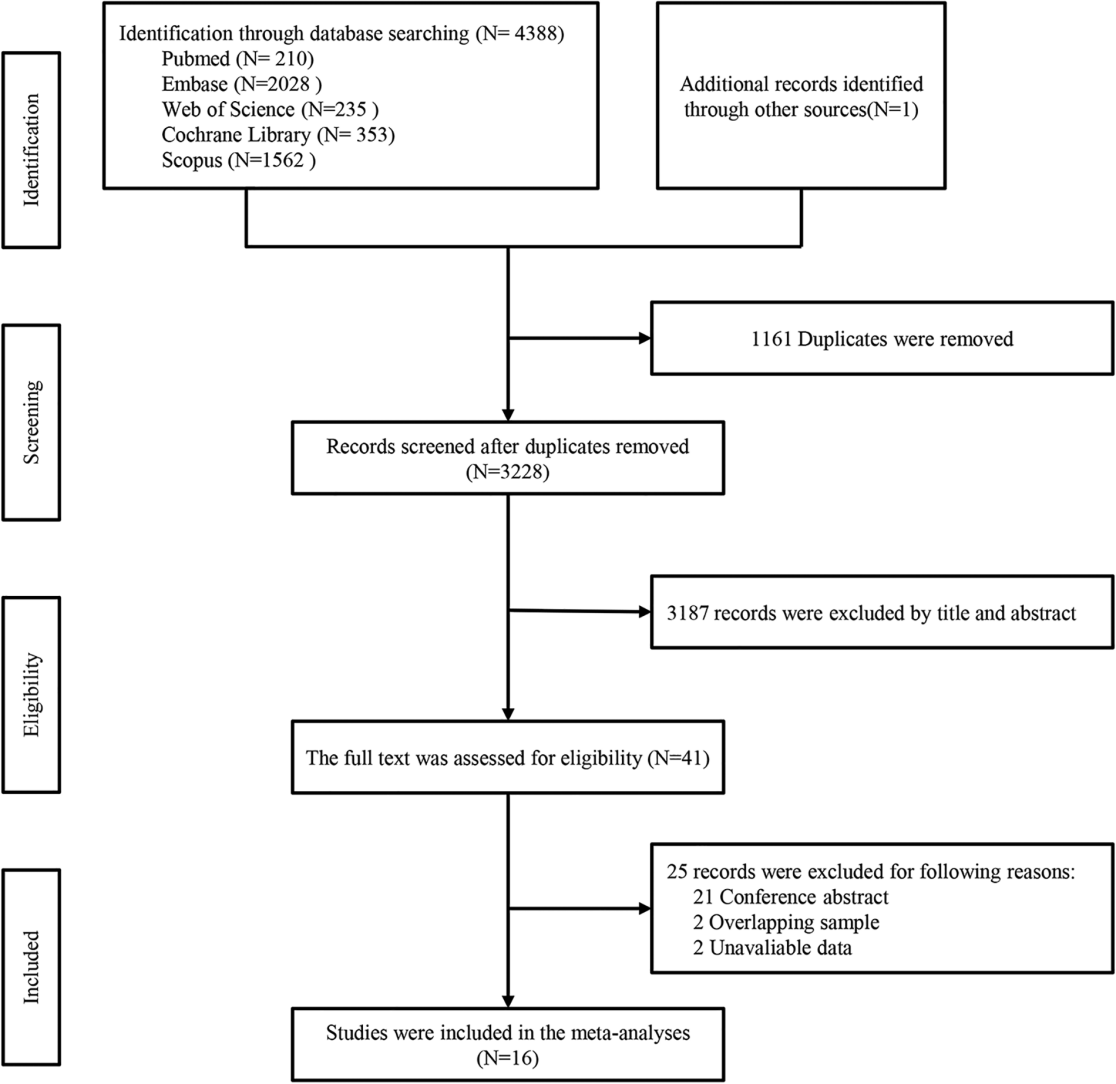
Flowchart of the systematic search and selection process.

The systematic literature search identified 4,389 related studies, with contributions from various databases: 210 from PubMed, 2,028 from Embase, 1,562 from Scopus, 353 from the Cochrane Library, and 235 from Web of Science. Following initial screening based on title and abstract, 3,227 articles underwent further evaluation. Subsequently, the complete texts of 43 articles were carefully reviewed. Among these, 21 conference abstracts and 2 articles (20, 21) with overlapping data were excluded, along with 2 studies (22, 23) lacking available data for analysis. Ultimately, sixteen articles, comprising a total of 20,796 patients, were included in the meta-analysis. These comprised 2 prospective cohort studies (24, 25), 13 retrospective cohort studies (26–38), and 1 prospective randomized study (39). Detailed study and patient characteristics are provided in Table 1. Among the included patients, 2,935 (14.1%) of 20,796 AF patients with HFpEF underwent CA, while 15,903 (76.5%) received medical therapy. Additionally, 794 (3.8%) of 20,796 AF patients with HFrEF and 1,164 (5.6%) of 20,796 AF patients without HF underwent CA. The study's flowchart is illustrated in Figure 1, while a comprehensive risk of bias analysis for the included studies is available in Supplementary Table S2 and Figure S1.

**Table 1:** Baseline characteristics of include studies and methodological assessment.

(A) CA vs. medical therapy for patients with HFpEF.													
Authors	Study period	Country	Study design	Patients (*n*)		Mean age (year)		Male, *N* (%)		BMI (Mean ± SD)		Follow-up period	Quality
				CA	Medical therapy	CA	Medical therapy	CA	Medical therapy	CA	Medical therapy	(Months)	Score
Arora et al.	2016–2017	USA	Retrospective	1053	15,795	73.0	76.0	569 (54.0)	6,980 (44.2)	NA	NA	12.0	8
Chieng et al.	2018–2021	Australia	Prospective	16	15	65.5	66.7	8 (50.0)	7 (46.7)	30.7 ± 6.0	32.4 ± 3.8	6.0	RCT
Fukui et al.	2014–2018	Japan	Retrospective	35	50	70.0	71.0	23 (65.7)	32 (64.0)	NA	NA	24.0	7
Rattka et al.	2013–2018	Germany	Retrospective	43	43	73.0	74.0	19 (44.2)	19 (44.2)	28.0 ± 3.1	28.3 ± 4.6	35.0	8
CA, catheter ablation; HFpEF, heart failure with preserved ejection fraction; BMI, body mass index; NA, not available; RCT, randomized controlled trial.													
(B) CA for patients with HFpEF vs. HFrEF.													
Authors	Study period	Country	Study design	Patients (*n*)		Mean age (year)		Male, *N* (%)		BMI (Mean ± SD)		Follow-up period	Quality
				HFpEF	HFrEF	HFpEF	HFrEF	HFpEF	HFrEF	HFpEF	HFrEF	(Months)	Score
Aldaas et al.	2009–2015	USA	Retrospective	51	40	66.2	66.7	31 (60.8)	32 (80.0)	29.8 ± 7.6	28.8 ± 5.2	40.0	7
Black-Maier et al.	2007–2013	USA	Retrospective	133	97	67.3	65.9	77 (57.9)	81 (83.5)	23.6 ± 3.5	30.1 ± 6.0	12.0	7
Cha et al.	2000–2007	USA	Retrospective	157	111	62.4	55.1	107 (68.0)	105 (95.0)	NA	NA	13.5	8
Eitel et al.	2007–2010	Germany	Prospective	333	188	65.4	65.0	220 (66.1)	145 (77.1)	NA	NA	12.0	8
Fujimoto et al.	2011–2014	Japan	Retrospective	451	98	67.0	64.6	305 (67.6)	82 (83.7)	23.6 ± 3.5	23.0 ± 3.4	60.0	8
Ichijo et al.	2010–2015	Japan	Prospective	55	51	64.0	60.0	44 (80.0)	41 (80.4)	25.5 ± 4.7	25.0 ± 4.3	32.0	7
Ishiguchi et al.	2009–2020	Japan	Retrospective	84	58	68.0	64.0	92 (65.0)	21 (36.0)	25.0 ± 4.0	23.0 ± 4.0	48.0	8
Qiao et al.	2018–2021	China	Retrospective	71	30	65.8	57.2	34 (47.9)	17 (56.7)	24.6 ± 3.1	24.0 ± 4.0	32.0	8
Yamauchi et al.	2013–2019	Japan	Retrospective	293	84	69.6	66.6	196 (66.9)	68 (81.0)	24.4 ± 3.9	24.0 ± 3.7	12.0	8
Chen et al.	2018–2021	China	Retrospective	101	37	66.3	68.4	60 (59.4)	23 (62.2)	23.1 ± 4.4	22.6 ± 4.6	23.1	7
CA, catheter ablation; HFpEF, heart failure with preserved ejection fraction; HFrEF, heart failure with reduced ejection fraction; BMI, Body mass index; NA, not available.													
(C) CA for patients with HFpEF vs. without HF.													
Authors	Study period	Country	Study design	Patients (*n*)		Mean age (year)		Male, *N* (%)		BMI (Mean ± SD)		Follow-up period	Quality
				HFpEF	Without HF	HFpEF	Without HF	HFpEF	Without HF	HFpEF	Without HF	(Months)	Score
Aldaas et al.	2009–2015	USA	Retrospective	51	456	66.2	64.1	31 (60.8)	307 (67.3)	29.8 ± 7.6	27.9 ± 4.5	50.9	7
Cha et al.	2000–2007	USA	Retrospective	157	100	62.4	52.1	107 (68.0)	75 (75.0)	NA	NA	13.2	8
Zylla et al.	2016–2019	Germany	Retrospective	24	78	72.2	63.6	20 (83.0)	22 (28.2)	23.1 ± 4.6	27.2 ± 4.2	12.0	8
Rattka et al.	2013–2017	Germany	Retrospective	35	150	69.0	64.0	14 (40.0)	87 (58.0)	29.0 ± 6.0	28.0 ± 5.0	29.0	8
Yamauchi et al.	2013–2019	Japan	Retrospective	293	125	69.6	64.2	196 (66.9)	109 (87.2)	24.4 ± 3.9	25.2 ± 3.6	12.0	8
Chen et al.	2018–2021	China	Retrospective	101	255	66.3	64.4	60 (59.4)	152 (59.6)	23.1 ± 4.4	22.8 ± 4.2	23.1	7

CA, catheter ablation; HFpEF, heart failure with preserved ejection fraction; HFrEF, heart failure with reduced ejection fraction; BMI, body mass index; NA, not available.

### 3.2 CA vs. medical therapy for patients with HFpEF

#### 3.2.1 Freedom From AF

**Figure 2:**
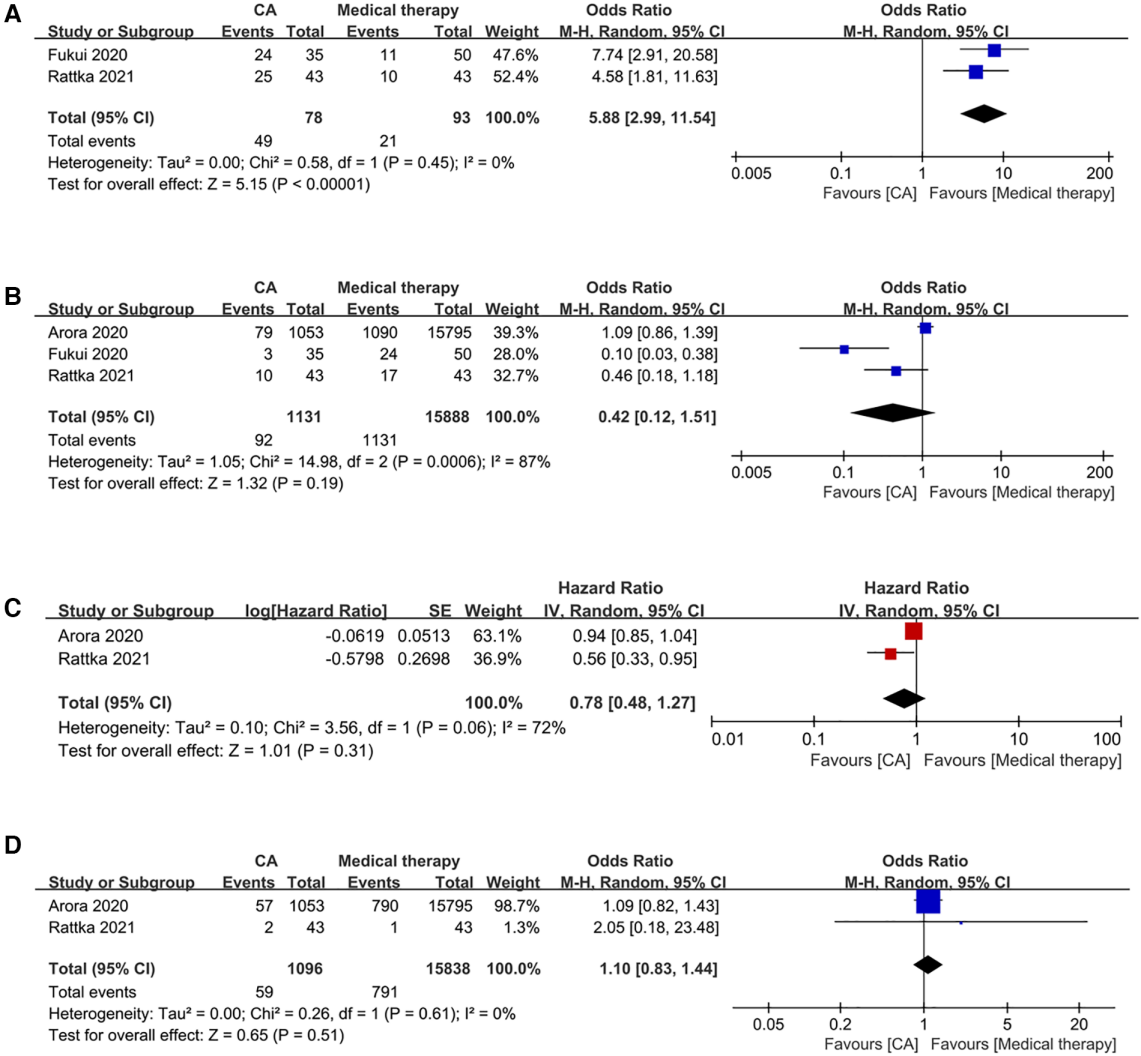
Forest plots of outcomes: (**A**) freedom from AF (**B**) HF admission (**C**) all-cause admission (**D**) all-cause mortality.

2 studies have documented this occurrence. Patients undergoing CA demonstrated a significantly higher freedom from AF compared to those receiving medical therapy (OR: 5.86; 95% CI: 2.99–11.49, *P* < 0.00001), with no observed heterogeneity (*I*^2^* *=* *0%; *P *= 0.45) (Figure 2A).

#### 3.2.2 HF admission

3 studies reported this event. Patients undergoing CA demonstrated comparable rates of HF admissions to those receiving medical therapy (OR: 0.42; 95% CI: 0.12–1.51, *P* = 0.19), with significant observed heterogeneity (*I*^2^ = 87%, *P *= 0.0006) (Figure 2B).

#### 3.2.3 All-cause admission

2 studies have provided data on this matter. Patients undergoing CA exhibited similar all-cause admission compared to those receiving medical therapy (HR: 0.78; 95% CI: 0.48–1.27, *P *= 0.31), with significant observed heterogeneity (*I*^2^ = 72%, *P *= 0.06) (Figure 2C).

#### 3.2.4 All-cause mortality

2 studies have reported data related to this event. Patients undergoing CA exhibited comparable all-cause mortality rates to those receiving medical therapy (OR: 1.10; 95% CI: 0.83–1.44, *P* = 0.51), with no discernible heterogeneity (*I*^2^ = 0%; *P *= 0.61) (Figure 2D).

#### 3.2.5 Sensitivity analysis

**Figure 3:**
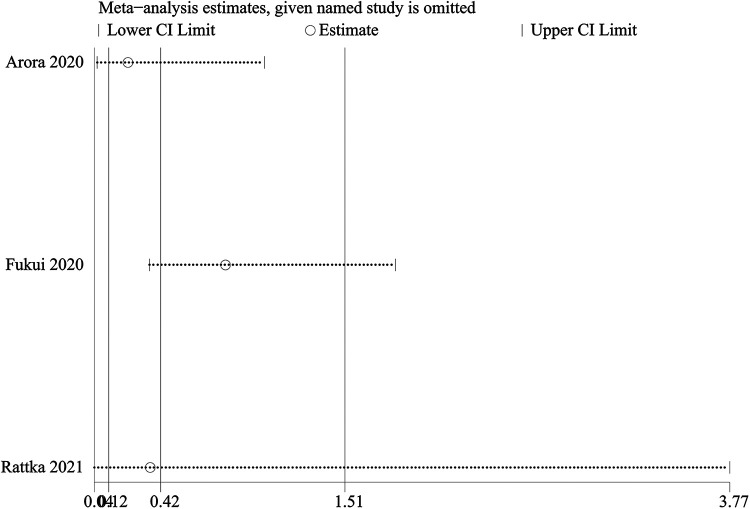
Sensitivity analysis of HF admission.

A one-way sensitivity analysis was performed to evaluate the influence of individual studies on the aggregated results, with the outcome consistently demonstrating coherence. Regardless of the exclusion of any individual study concerning HF admission, the analysis revealed that the recalculated combined odds ratio remained consistently stable (Figure 3).

### 3.3 CA for patients with HFpEF vs. HFrEF

#### 3.3.1 Freedom from AF

**Figure 4:**
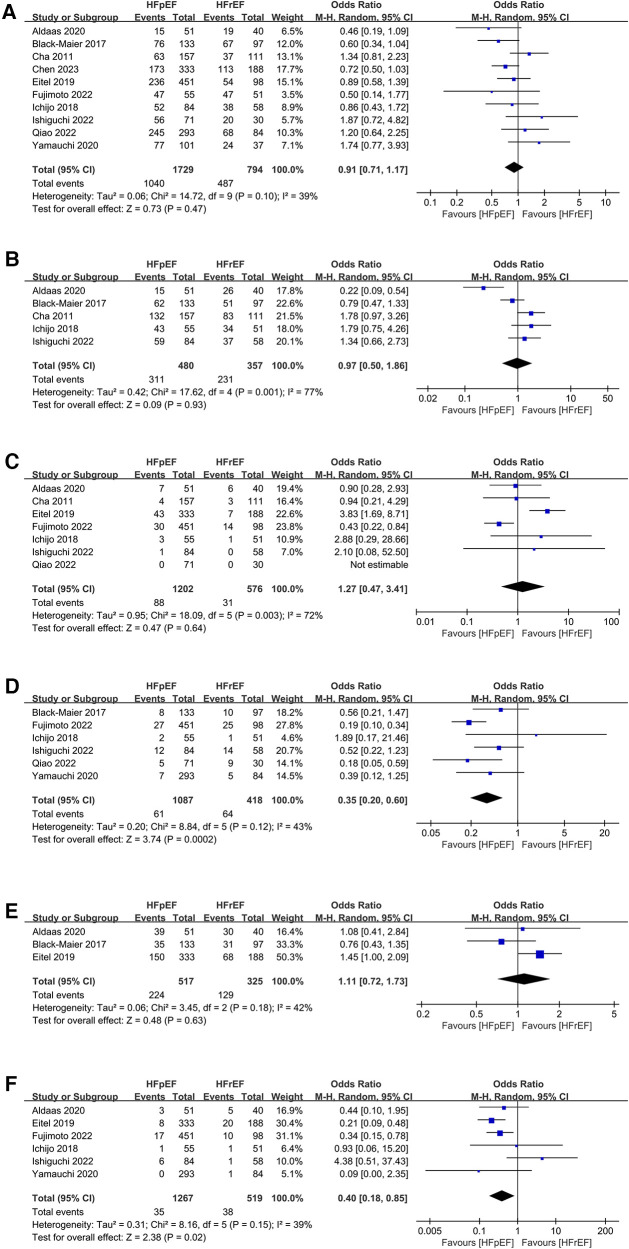
Forest plots of outcomes: (**A**) freedom from AF (**B**) AAD-free AF elimination (**C**) total complications (**D**) HF admission (**E**) all-cause admission (**F**) all-cause mortality.

**Figure 5:**
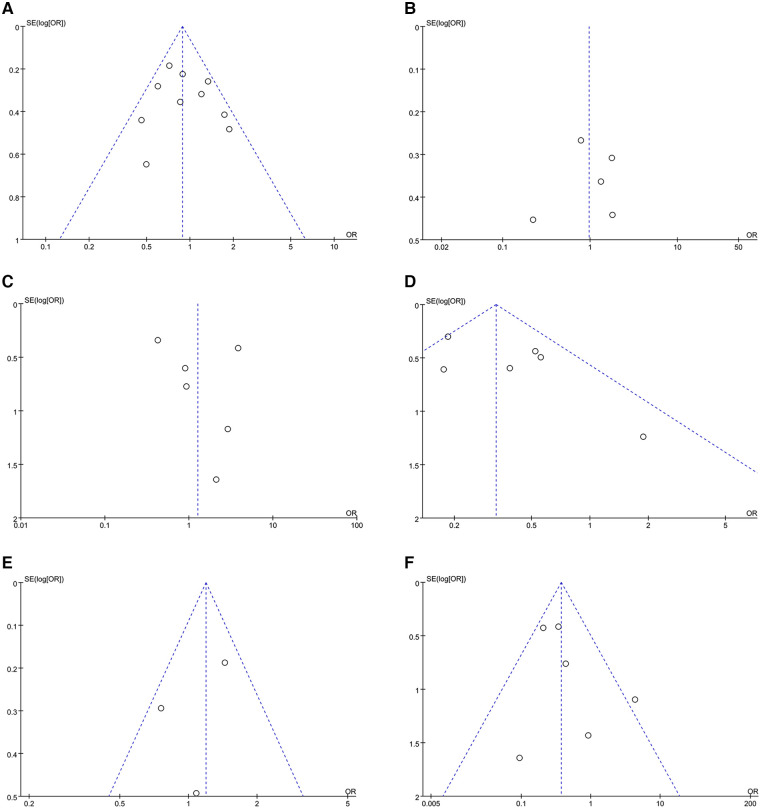
Funnel plots of outcomes: (**A**) freedom from AF (**B**) AAD-free AF elimination (**C**) total complications (**D**) HF admission (**E**) all-cause admission (**F**) all-cause mortality.

Synthesized from a compilation of 10 studies, the data on freedom from AF involve a total of 2,523 patients, with 1,729 having HFpEF and 794 with HFrEF. No significant difference in freedom from AF were observed (OR: 0.91; 95% CI: 0.71, 1.17, *P *= 0.47), with no statistically heterogeneity observed (*I*^2^ = 39%; Figure 4A). While a slight visual indication of publication bias was noted in Figure 5A, Egger's test did not reveal any statistically significant evidence of publication bias (*P* = 0.650).

#### 3.3.2 AAD-free AF elimination

Analysis of this event was conducted among 5 studies involving 837 patients (480 HFpEF vs. 357 HFrEF). Patients with HFpEF exhibited comparable AAD-Free AF Elimination rates to those with HFrEF (OR: 0.97; 95% CI: 0.50–1.86, *P *= 0.93), with notable heterogeneity (*I*^2^ = 77%, *P *= 0.001) (Figure 4B). A slight visual (Figure 5B) evidence of publication bias was observed, however, Egger's test did not show a statistically significant publication bias (*P *= 0.724). Subgroup analysis was subsequently performed based on exposed group population (HFrEF, HFrEF + HFmrEF), study design (prospective, retrospective), follow-up (<24months, ≥24months), and region (Asia, Europe, America), did not reveal significant changes in AAD-free AF elimination associated with these factors (Table 2).

**Table 2:** Subgroup analysis.

(A). Subgroup analysis of CA for patients with HFpEF vs. HFrEF.																				
	freedom from AF				HF admission				Total complication				All-cause mortality				AAD-free AF elimination			
	Study	OR [95%CI]	*P* value	*I^2^*	Study	OR [95%CI]	*P* value	*I^2^*	Study	OR [95%CI]	*P* value	*I^2^*	Study	OR [95%CI]	*P* value	*I^2^*	Study	OR [95%CI]	*P* value	*I^2^*
Total	10	0.91 [0.71, 1.17]	0.47	39%	6	0.35 [0.20, 0.60]	0.0002	43%	6	1.27 [0.47, 3.41]	0.64	72%	6	0.40 [0.18, 0.85]	0.02	39%	5	0.97 [0.50, 1.86]	0.93	77%
Population																				
HFrEF	7	0.79 [0.62,1.01]	0.06	23%	3	0.40 [0.13, 1.23]	0.11	68%	5	1.23 [0.43, 3.54]	0.7	78%	4	0.29 [0.17, 0.50]	<0.00001	0%	4	0.89 [0.39, 2.03]	0.78	82%
HFrEF + HFmrEF	3	1.47 [0.95, 2.27]	0.09	0%	3	0.37 [0.20, 0.68]	0.001	5%	1	2.10 [0.08, 52.50]	0.65	/	2	1.47 [0.36, 6.02]	0.59	74%	1	1.34 [0.66, 2.73]	0.42	/
Study design																				
Prospective	2	0.88 [0.61, 1.28]	0.51	0%	1	1.89 [0.17, 21.46]	0.61	/	2	3.71 [1.71, 8.04]	0.0009	0%	2	0.24 [0.10, 0.54]	0.0006	1%	1	1.79 [0.75, 4.26]	0.19	/
Retrospective	8	0.93 [0.66, 1.30]	0.66	52%	5	0.32 [0.19, 0.54]	<0.00001	40%	4	0.58 [0.34, 0.99]	0.05	0%	4	0.52 [0.16, 1.66]	0.27	49%	4	0.84 [0.39, 1.80]	0.66	81%
Follow-up																				
<24months	5	0.92 [0.66, 1.27]	0.60	53%	2	0.48 [0.23, 1.01]	0.05	0%	1	3.83 [1.69, 8.71]	0.001	/	2	0.20 [0.09, 0.44]	<0.0001	0%	2	1.17 [0.52, 2.60]	0.70	75%
≥24months	5	0.90 [0.56, 1.43]	0.65	36%	4	0.31 [0.14, 0.68]	0.003	56%	5	0.65 [0.37, 1.13]	0.13	4%	4	0.64 [0.22, 1.84]	0.41	43%	3	0.84 [0.25, 2.76]	0.75	85%
Region																				
Asia	6	1.00 [0.70, 1.43]	1.00	37%	5	0.31 [0.17, 0.58]	0.0003	44%	3	0.57 [0.31, 1.08]	0.08	39%	4	0.63 [0.14, 2.73]	0.54	52%	4	0.29 [0.19, 0.45]	0.73	0%
Europe	1	0.89 [0.58, 1.39]	0.62	/	/	/	/	/	1	3.83 [1.69, 8.71]	0.001	/	1	0.21 [0.09, 0.48]	0.0002	/	0	/	/	/
America	3	0.76 [0.39, 1.45]	0.40	70%	1	0.56 [0.21, 1.47]	0.24	/	2	0.92 [0.36, 2.32]	0.85	0%	1	0.44 [0.10, 1.95]	0.28	/	1	0.56 [0.21, 1.47]	0.24	/
AF, atrial fibrillation; CA, catheter ablation; HF, heart failure; HFpEF, heart failure with preserved ejection fraction; HFrEF, heart failure with reduced ejection fraction; OR, odds ratio; CI, confidence interval; AAD-free AF elimination, antiarrhythmic drugs free atrial fibrillation elimination.																				

(B) Subgroup analysis of CA for patients with HFpEF vs. without HF.				
Subgroup	Freedom from AF			
	Study	OR [95% CI]	*P* value	*I^2^*
Total	6	0.70 [0.48, 1.02]	0.06	41%
Follow-up				
<24 months	2	0.77 [0.41, 1.47]	0.43	52%
≥24 months	4	0.64 [0.37, 1.10]	0.11	51%
Region				
Asia	2	0.97 [0.54, 1.71]	0.90	29%
Europe	2	0.37 [0.20, 0.67]	0.001	0%
America	2	0.81 [0.53, 1.24]	0.34	0%

CA, catheter ablation; AF, atrial fibrillation; HF, heart failure; HFpEF, heart failure with preserved ejection fraction; OR, odds ratio; CI, confidence interval.

#### 3.3.3 Total complications

Analysis of total complications included 6 studies, encompassing 1,778 patients (1,202 HFpEF and 576 HFrEF) totally. The findings revealed no significant difference between groups (OR: 1.27; 95% CI: 0.47–3.41, *P *= 0.64). However, a notable heterogeneity exist (*I*^2^ = 72%, *P *= 0.003) (Figure 4C). While a slight visual indication of publication bias was apparent (Figure 5C), Egger's test did not detect any statistically significant publication bias (*P *= 0.596). To address the observed heterogeneity, subgroup analyses were conducted based on study design. The prospective analysis, which included two studies, revealed a significantly higher incidence of total complications (OR: 3.71; 95% CI: 1.71–8.04, *P *= 0.0009). Conversely, the retrospective analysis, encompassing three articles, demonstrated a comparable incidence of total complications (OR: .58; 95% CI: 0.34–0.99, *P *= 0.05) (Table 2).

#### 3.3.4 HF admission

Data on HF admission were synthesized from 6 studies, encompassing 1,505 patients (1,087 HFpEF vs. 418 HFrEF). Patients with HFpEF exhibited a lower HF admission (OR: 0.35; 95% CI: 0.20, 0.60; *P *= 0.0002), with no statistically heterogeneity (*I*^2^ = 43%; Figure 4D). Funnel plots indicated a slight presence of publication bias (Figure 5D), whereas Egger's test did not detect any publication bias (*P *= 0.177).

#### 3.3.5 All-cause admission

Analysis of all-cause admission was conducted in 3 studies with 842 patients (517 HFpEF vs. 325 HFrEF). The evidence synthesis observed similar rate of all-cause admission in both groups (OR: 1.11; 95% CI: 0.72, 1.73; *P *= 0.63), with no statistically heterogeneity (*I*^2 ^= 42%; Figure 4E). While a slight visual indication of publication bias was observed (Figure 5E), Egger's test did not detect publication bias (*P* = 0.600).

#### 3.3.6 All-cause mortality

Analysis of all-cause mortality was conducted in 6 studies with 1,786 patients (1,267 HFpEF vs. 519 HFrEF). Pooled analysis indicated that CA in HFpEF resulted in a significant reduction in all-cause mortality (OR: 0.40; 95% CI: 0.18, 0.85; *P* = 0.02), with no statistically heterogeneity (*I*^2 ^= 39%; Figure 4F). While a slight indication of publication bias was observed in Figure 5F, Egger's test did not identify publication bias (*P *= 0.364).

#### 3.3.7 Sensitivity analysis

**Figure 6:**
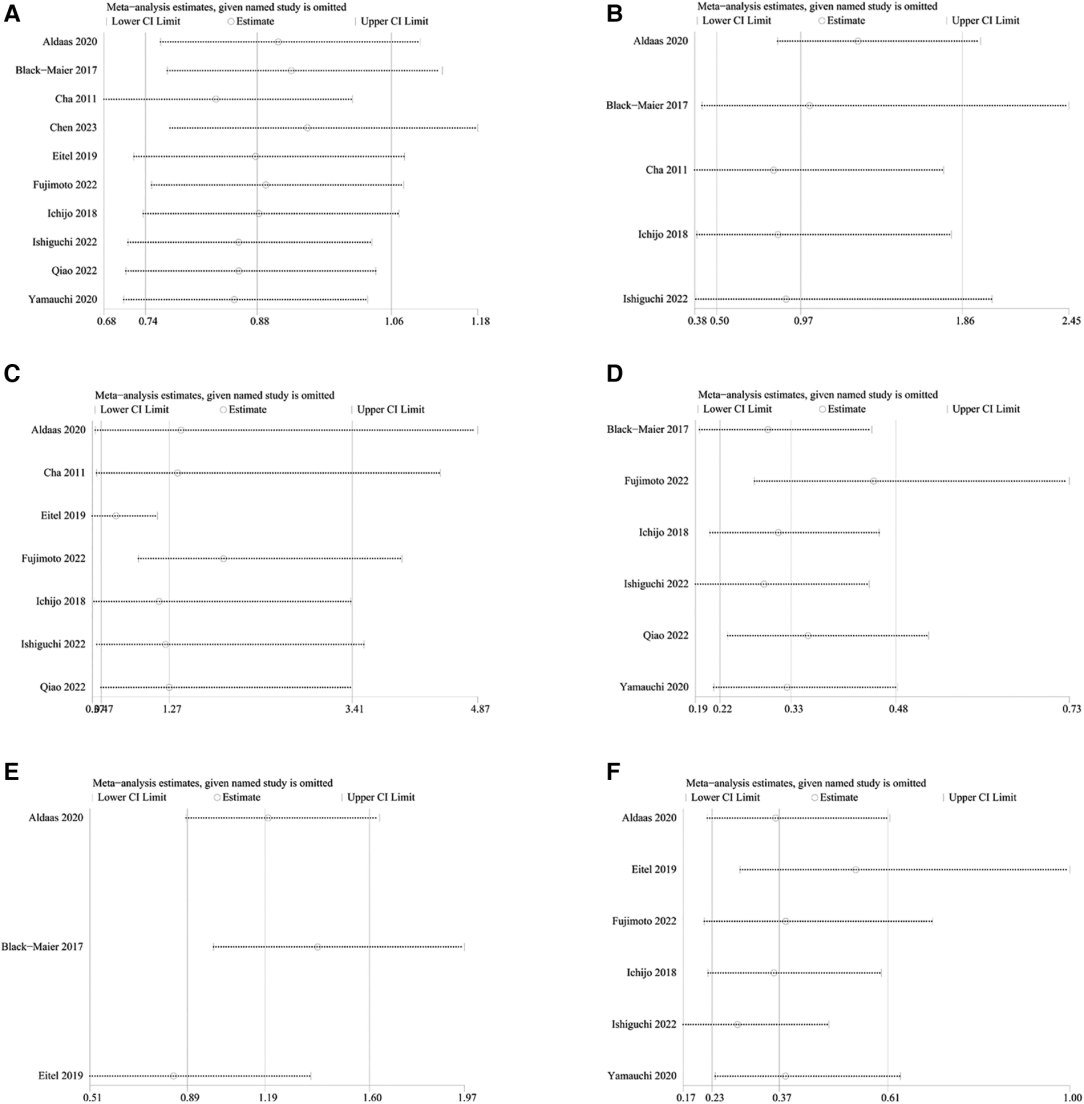
Sensitivity analysis of outcomes: (**A**) freedom from AF (**B**) AAD-free AF elimination (**C**) total complications (**D**) HF admission (**E**) all-cause admission (**F**) all-cause mortality.

One-way sensitivity analyses were conducted to evaluate the influence of individual studies on different outcomes, including AF recurrence, AAD-free AF elimination, total complications, HF admission, all-cause admission, and all-cause mortality. This evaluation aimed to determine the consistency of results by systematically removing each study. Sensitivity analyses indicated that the findings remained consistent across all outcomes, as illustrated in Figures 6A–F.

### 3.4 CA for patients with HFpEF vs. without HF

#### 3.4.1 Freedom from AF

**Figure 7:**
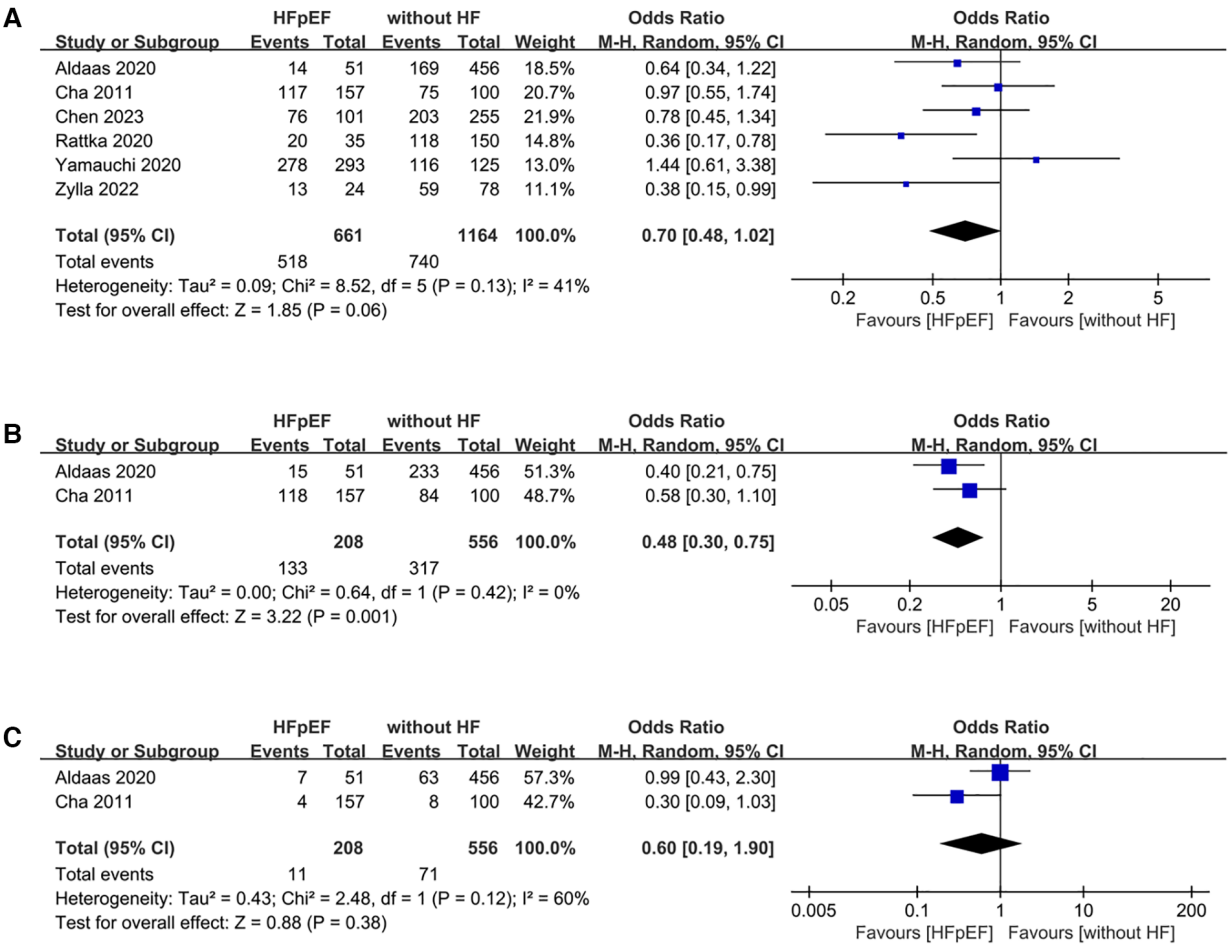
Forest plots of outcomes: (**A**) freedom from AF (**B**) AAD-free AF elimination (**C**) total complications.

**Figure 8:**
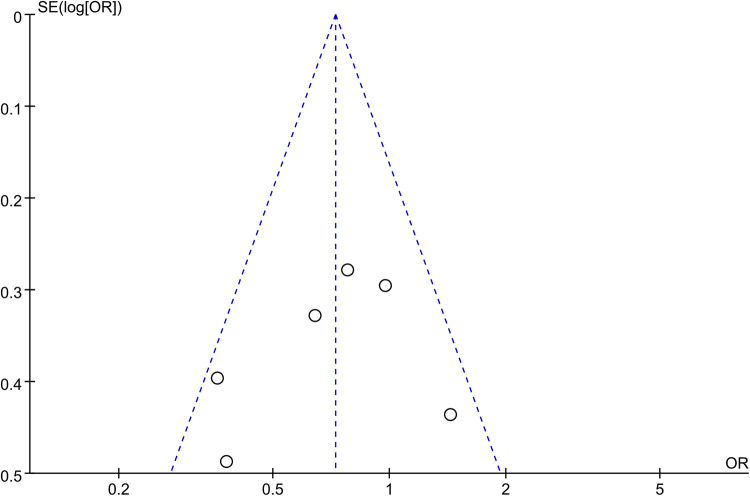
Funnel plots of freedom from AF.

Analysis of freedom from AF encompassed data from 6 studies, involving a total of 1,825 patients (661 HFpEF vs. 1,164 HF). The HFpEF group exhibited significantly lower freedom from AF (OR: 0.70; 95% CI: 0.48, 1.02; *P* = 0.06), with no statistically heterogeneity (*I*^2^ = 41%, *P* = 0.13) (Figure 7A). While a slight indication of publication bias shown in Figure 8, Egger's test did not detect any publication bias (*P* = 0.492).

#### 3.4.2 AAD-free AF elimination

The analysis of AAD-free AF elimination was conducted across 2 studies involving 764 patients (208 HFpEF vs. 556 without HF). Evidence synthesis revealed a lower AAD-free AF elimination in the HFpEF group (OR: 0.48; 95% CI: 0.30, 0.75; *P* = 0.001). No heterogeneity was observed (*I*^2^ = 0%; *P* = 0.42) (Figure 7B).

#### 3.4.3 Total complications

Total complications was conducted across 2 studies involving 764 patients (208 HFpEF vs. 556 without HF). The evidence synthesis observed similar rate of total complications in both groups (OR: 0.60; 95% CI: 0.19, 1.90; *P* = 0.38). However, statistically significant heterogeneity existed (*I*^2 ^= 60%, *P *= 0.12) (Figure 7C).

#### 3.4.4 Sensitivity analysis

**Figure 9:**
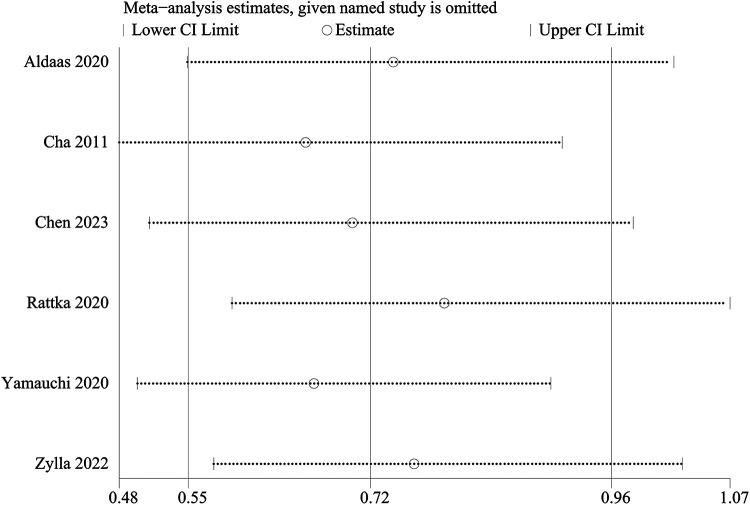
Sensitivity analysis of freedom from AF.

During sensitivity analysis, the results remained consistent even after excluding each individual study (Figure 9).

## 4 Discussion

Presently, research on HF and AF is increasingly focusing on HFpEF and AF. Not only is the prevalence of HFpEF gradually increasing, surpassing that of HFrEF, but also the incidence of AF in HFpEF is higher than in HFrEF. This coexistence of HFpEF and AF poses a greater risk. CA is acknowledged as an effective treatment for AF and AF with HFrEF. However, the potential benefits of CA for patients with AF and HFpEF remain uncertain.

To evaluate the outcomes of CA in patients with AF and HFpEF, our meta-analysis systematically synthesized findings from sixteen published studies. The key findings are outlined below: (a) CA significantly improves freedom from AF in HFpEF patients, while demonstrating similar rates of HF admission, all-cause admission, and all-cause mortality compared to medical therapy. (b) Patients with HFpEF derive comparable benefits from CA as HFrEF patients, with lower rates of HF admission and all-cause mortality. Additionally, HFpEF patients experience similar rates of freedom from AF, AAD-free AF elimination, total complications, and all-cause admission compared to HFrEF patients. (c) Although the AAD-free AF elimination was lower after CA in HFpEF than in those without HF, patients with HFpEF benefited from CA similarly to those without HF, with a similar freedom from AF and total complications.

In achieving rhythm control among AF patients with HFpEF, our study, consistent with the findings of Gu et al. (40), demonstrates that CA surpasses medical therapy. Aligned with Aldaas et al.'s study (41), our systematic meta-analysis underscores that, in patients with HFpEF, CA yields results in terms of freedom from AF comparable to those observed in patients with HFrEF. However, compared to patients without HF, those with HFpEF exhibit lower freedom from AF. It is noteworthy that risk factors for HFpEF, such as increased left atrial diameter, substrate remodeling, arterial hypertension, and female sex, are also associated with lower freedom from AF rates of AF after CA (42).

Previous studies have demonstrated the safety of performing CA in patients with AF, even in the context of HFpEF (40, 43). In our study, the total complications are comparable among HFpEF and HFrEF, as well as between HFpEF and those without HF. Within the HFpEF and HFrEF groups, a higher incidence of overall complications was observed in prospective studies, according to the subgroup analysis based on study design. This observation may be attributed to the inclusion of only 2 studies in the prospective design, underscoring the need for further exploration through additional studies.

Reaffirming previous findings (41), our study contributes additional evidence supporting the association between CA involvement in AF patients with HFpEF and a notably diminished risk of all-cause mortality when compared to individuals with HFrEF. Furthermore, our findings also reveal that CA in AF patients with HFpEF results in a lower rate of HF admission compared to those with HFrEF. However, the reason for this difference is not yet clear. Since our study was a *post hoc* analysis, further insights will be provided by large-scale prospective studies.

CA for AF involves various energy modalities, primarily including radiofrequency ablation and cryoballoon ablation (10). Previous studies have demonstrated that both radiofrequency ablation and cryoballoon ablation are safe and effective for AF patients. However, some studies have indicated that patients undergoing radiofrequency ablation may experience a higher incidence of long-term pulmonary hypertension (44). The majority of studies included in this analysis did not specify the type of energy used for CA. Therefore, more detailed and well-designed studies are needed to elucidate the long-term complications associated with different energy modalities in patients with AF and HFpEF.

The correlation between AF and HF arises from complex interplay of pathophysiological mechanisms. The risk factors for AF and HF exhibit significant overlap, for example, age, obesity, smoking, alcohol consumption, hypertension, diabetes, chronic kidney disease, obstructive sleep apnea, and coronary artery disease (26, 45–47). Moreover, they have a mutual tendency to precipitate each other, and over time, these conditions may perpetuate or worsen one another in complementary ways.

These identified risk factors contribute to the alteration of both atrial and ventricular function, primarily through inducing inflammatory processes, fibrotic changes, hemodynamic stress, and ischemic events. Consequently, they lead to extensive structural, mechanical, and electrophysiological remodeling (48, 49). In the context of AF, it is important to recognize its dual role in the development of HF. Initially, AF can play a direct role in advancing or intensifying HF by compromising atrial contraction and disrupting the coordination between atrial and ventricular contractions. This often leads to diminished cardiac output, increased ventricular filling pressure, and activation of neurohormonal pathways. Conversely, HF itself can predispose individuals to the development of AF through various mechanisms. Elevated filling pressures in HF can induce mechanical stress and structural remodeling within the atria, fostering a substrate conducive to the initiation and perpetuation of AF. Furthermore, alterations in the electrophysiological properties of atrial tissue, disturbances in calcium handling, and the activation of neurohormonal and adrenergic pathways further contribute to the onset of AF in individuals with HF (50). Additionally, histopathological studies have elucidated that interstitial fibrosis, increased intercellular gaps, myofibrillar loss, and reduced nuclear density contribute to atrial structural remodeling, which is associated with the persistence of atrial fibrillation and its recurrence post-ablation (51, 52).

Observational studies suggest that restoring sinus rhythm via CA may modulate the disease progression of AF and HFpEF by interrupting the interdependent perpetuation of AF and HFpEF. CA procedures have shown significant results, such as decreased natriuretic peptide levels, reduced left atrial dimensions, lowered pulmonary capillary wedge pressure during exercise and at rest, increased peak cardiac output, improved New York Heart Association functional capacity, and potential partial resolution of HFpEF (36, 37, 53, 54). In line with a causal relationship, the resolution of HFpEF exhibited a strong correlation with the absence of arrhythmia recurrence following ablation.

Our meta-analysis exhibited several limitations. Firstly, only one randomized controlled trial was included in the analysis. Secondly, the number of studies within each category of analysis is relatively small. In particular, in the category comparing CA to medical therapy for patients with HFpEF, only two or three studies were included. Thirdly, the studies examined did not provide separate data for paroxysmal atrial fibrillation and persistent atrial fibrillation, thus preventing subgroup analyses for these conditions. Fourthly, most of the studies examined did not specify the type of energy used in CA, i.e., thermal (radiofrequency ablation and cryoballoon ablation) or non-thermal (pulsed field ablation), thus precluding further analysis of complications associated with different energy types. Fifthly, the median follow-up duration of the included studies was 23.6 months, highlighting the need for further research to assess the longer-term efficacy of CA. Lastly, publication bias could not be entirely ruled out, as only published data were included, contributing to potential bias. Therefore, it is imperative to conduct more well-designed and large-scale randomized controlled trials to validate these findings.

## 5 Conclusions

This meta-analysis comprehensively evaluated the efficacy and safety of CA in patients with AF and HFpEF. The results of the analysis revealed that CA demonstrates comparable safety and effectiveness to medical therapy. Moreover, when compared to patients with HFrEF, CA for HFpEF not only shows similar effectiveness but also leads to reduced rates of HF hospitalizations and all-cause mortality. Despite a lower efficacy of CA in treating HFpEF patients compared to those without HF, its safety profile remains comparable. These findings suggest that CA could serve as an effective and safe strategy for managing patients with AF and HFpEF.

## Data Availability

The original contributions presented in the study are included in the article/Supplementary Material, further inquiries can be directed to the corresponding author.
