# Supplementary material for: Efficacy and safety of catheter ablation for atrial fibrillation in patients with heart failure with preserved ejection fraction: a systematic review and meta-analysis
Source: Front Cardiovasc Med. 2024 Jul 25;11:1423147. doi: 10.3389/fcvm.2024.1423147 (PMC11306038; doi:10.3389/fcvm.2024.1423147)

**Supplementary materials**

**Table S1 Literature search strategy**

**1.Pubmed**

| Search number | Query |
| --- | --- |
| #1 | Atrial Fibrillation[MeSH Terms] |
| #2 | ((((((((((((((((((((((((Atrial Fibrillations[Title/Abstract]) OR (Fibrillation, Atrial[Title/Abstract])) OR (Fibrillations, Atrial[Title/Abstract])) OR (Auricular Fibrillation[Title/Abstract])) OR (Auricular Fibrillations[Title/Abstract])) OR (Fibrillation, Auricular[Title/Abstract])) OR (Fibrillations, Auricular[Title/Abstract])) OR (Persistent Atrial Fibrillation[Title/Abstract])) OR (Atrial Fibrillation, Persistent[Title/Abstract])) OR (Atrial Fibrillations, Persistent[Title/Abstract])) OR (Fibrillation, Persistent Atrial[Title/Abstract])) OR (Fibrillations, Persistent Atrial[Title/Abstract])) OR (Persistent Atrial Fibrillations[Title/Abstract])) OR (Familial Atrial Fibrillation[Title/Abstract])) OR (Atrial Fibrillation, Familial[Title/Abstract])) OR (Atrial Fibrillations, Familial[Title/Abstract])) OR (Familial Atrial Fibrillations[Title/Abstract])) OR (Fibrillation, Familial Atrial[Title/Abstract])) OR (Fibrillations, Familial Atrial[Title/Abstract])) OR (Paroxysmal Atrial Fibrillation[Title/Abstract])) OR (Atrial Fibrillation, Paroxysmal[Title/Abstract])) OR (Atrial Fibrillations, Paroxysmal[Title/Abstract])) OR (Fibrillation, Paroxysmal Atrial[Title/Abstract])) OR (Fibrillations, Paroxysmal Atrial[Title/Abstract])) OR (Paroxysmal Atrial Fibrillations[Title/Abstract]) |
| #3 | #1 or #2 |
| #4 | Heart Failure[MeSH Terms] |
| #5 | (((((((((((((Cardiac Failure[Title/Abstract]) OR (Heart Decompensation[Title/Abstract])) OR (Decompensation, Heart[Title/Abstract])) OR (Heart Failure, Right-Sided[Title/Abstract])) OR (Heart Failure, Right Sided[Title/Abstract])) OR (Right-Sided Heart Failure[Title/Abstract])) OR (Right Sided Heart Failure[Title/Abstract])) OR (Myocardial Failure[Title/Abstract])) OR (Congestive Heart Failure[Title/Abstract])) OR (Heart Failure, Congestive[Title/Abstract])) OR (Heart Failure, Left-Sided[Title/Abstract])) OR (Heart Failure, Left Sided[Title/Abstract])) OR (Left-Sided Heart Failure[Title/Abstract])) OR (Left Sided Heart Failure[Title/Abstract]) |
| #6 | Heart Failure, Diastolic[MeSH Terms] |
| #7 | (((((Diastolic Heart Failures[Title/Abstract]) OR (Heart Failure, Preserved Ejection Fraction[Title/Abstract])) OR (Heart Failure, Normal Ejection Fraction[Title/Abstract])) OR (Diastolic Heart Failure[Title/Abstract])) OR (heart failure with preserved ejection fraction[Title/Abstract])) OR (HFpEF[Title/Abstract]) |
| #8 | #4 or #5 or #6 or #7 |
| #9 | Catheter ablation[MeSH Terms] |
| #10 | (((((((((((((((((((((Ablation, Catheter[Title/Abstract]) OR (Catheter Ablation, Transvenous[Title/Abstract])) OR (Transvenous Catheter Ablation[Title/Abstract])) OR (Ablation, Transvenous Catheter[Title/Abstract])) OR (Catheter Ablation, Electric[Title/Abstract])) OR (Electrical Catheter Ablation[Title/Abstract])) OR (Catheter Ablation, Electrical[Title/Abstract])) OR (Ablation, Electrical Catheter[Title/Abstract])) OR (Electric Catheter Ablation[Title/Abstract])) OR (Ablation, Electric Catheter[Title/Abstract])) OR (Ablation, Transvenous Electric[Title/Abstract])) OR (Electric Ablation, Transvenous[Title/Abstract])) OR (Transvenous Electric Ablation[Title/Abstract])) OR (Ablation, Transvenous Electrical[Title/Abstract])) OR (Electrical Ablation, Transvenous[Title/Abstract])) OR (Transvenous Electrical Ablation[Title/Abstract])) OR (Catheter Ablation, Radiofrequency[Title/Abstract])) OR (Radiofrequency Catheter Ablation[Title/Abstract])) OR (Ablation, Radiofrequency Catheter[Title/Abstract])) OR (Catheter Ablation, Percutaneous[Title/Abstract])) OR (Percutaneous Catheter Ablation[Title/Abstract])) OR (Ablation, Percutaneous Catheter[Title/Abstract]) |
| #11 | #9 or #10 |
| #12 | randomized controlled trial OR randomized controlled trial OR RCT OR clinical trial OR cohort OR case-control OR clinical study |
| #13 | #3 and #8 and #11 and #12 |

**2.the Cochrane library**

| Search number | Query |
| --- | --- |
| #1 | MeSH descriptor: [Atrial Fibrillation] explode all trees |
| #2 | (Atrial Fibrillations):ab,ti,kw OR (Fibrillation, Atrial):ab,ti,kw OR (Fibrillations, Atrial):ab,ti,kw OR (Auricular Fibrillation):ab,ti,kw OR (Auricular Fibrillations):ab,ti,kw OR (Fibrillation, Auricular):ab,ti,kw OR (Fibrillations, Auricular):ab,ti,kw OR (Persistent Atrial Fibrillation):ab,ti,kw OR (Atrial Fibrillation, Persistent):ab,ti,kw OR (Atrial Fibrillations, Persistent):ab,ti,kw OR (Fibrillation, Persistent Atrial):ab,ti,kw OR (Fibrillations, Persistent Atrial):ab,ti,kw OR (Persistent Atrial Fibrillations):ab,ti,kw OR (Familial Atrial Fibrillation):ab,ti,kw OR (Atrial Fibrillation, Familial):ab,ti,kw OR (Atrial Fibrillations, Familial):ab,ti,kw OR (Familial Atrial Fibrillations):ab,ti,kw OR (Fibrillation, Familial Atrial):ab,ti,kw OR (Fibrillations, Familial Atrial):ab,ti,kw OR (Paroxysmal Atrial Fibrillation):ab,ti,kw OR (Atrial Fibrillation, Paroxysmal):ab,ti,kw OR (Atrial Fibrillations, Paroxysmal):ab,ti,kw OR (Fibrillation, Paroxysmal Atrial):ab,ti,kw OR (Fibrillations, Paroxysmal Atrial):ab,ti,kw OR (Paroxysmal Atrial Fibrillations):ab,ti,kw |
| #3 | MeSH descriptor: [Heart Failure] explode all trees |
| #4 | (Cardiac Failure):ab,ti,kw OR (Heart Decompensation):ab,ti,kw OR (Decompensation, Heart):ab,ti,kw OR (Heart Failure, Right-Sided):ab,ti,kw OR (Heart Failure, Right Sided):ab,ti,kw OR (Right-Sided Heart Failure):ab,ti,kw OR (Right Sided Heart Failure):ab,ti,kw OR (Myocardial Failure):ab,ti,kw OR (Congestive Heart Failure):ab,ti,kw OR (Heart Failure, Congestive):ab,ti,kw OR (Heart Failure, Left-Sided):ab,ti,kw OR (Heart Failure, Left Sided):ab,ti,kw OR (Left-Sided Heart Failure):ab,ti,kw OR (Left Sided Heart Failure):ab,ti,kw OR (heart failure with preserved ejection fraction):ab,ti,kw OR (HFpEF):ab,ti,kw OR (Heart Failure, Diastolic):ab,ti,kw OR (Diastolic Heart Failures):ab,ti,kw OR (Heart Failure, Preserved Ejection Fraction):ab,ti,kw OR (Heart Failure, Normal Ejection Fraction):ab,ti,kw OR (Diastolic Heart Failure):ab,ti,kw |
| #5 | MeSH descriptor: [Catheter Ablation] explode all trees |
| #6 | (Ablation, Catheter):ab,ti,kw OR (Catheter Ablation, Transvenous):ab,ti,kw OR (Transvenous Catheter Ablation):ab,ti,kw OR (Ablation, Transvenous Catheter):ab,ti,kw OR (Catheter Ablation, Electric):ab,ti,kw OR (Electrical Catheter Ablation):ab,ti,kw OR (Catheter Ablation, Electrical):ab,ti,kw OR (Ablation, Electrical Catheter):ab,ti,kw OR (Electric Catheter Ablation):ab,ti,kw OR (Ablation, Electric Catheter):ab,ti,kw OR (Ablation, Transvenous Electric):ab,ti,kw OR (Electric Ablation, Transvenous):ab,ti,kw OR (Transvenous Electric Ablation):ab,ti,kw OR (Ablation, Transvenous Electrical):ab,ti,kw OR (Electrical Ablation, Transvenous):ab,ti,kw OR (Transvenous Electrical Ablation):ab,ti,kw OR (Catheter Ablation, Radiofrequency):ab,ti,kw OR (Radiofrequency Catheter Ablation):ab,ti,kw OR (Ablation, Radiofrequency Catheter):ab,ti,kw OR (Catheter Ablation, Percutaneous):ab,ti,kw OR (Percutaneous Catheter Ablation):ab,ti,kw OR (Ablation, Percutaneous Catheter):ab,ti,kw |
| #7 | (randomized controlled trial OR randomized controlled trial OR RCT OR clinical trial OR cohort OR case-control OR clinical study) |
| #8 | (#1 OR #2) AND (#3 OR #4) AND (#5 OR #6) AND #7 |

**3.Embase**

| Search number | Query |
| --- | --- |
| #1 | 'atrial fibrillation'/exp |
| #2 | 'atrium fibrillation':ab,ti OR 'auricular fibrilation':ab,ti OR 'auricular fibrillation':ab,ti OR 'cardiac atrial fibrillation':ab,ti OR 'cardiac atrium fibrillation':ab,ti OR 'fibrillation, heart atrium':ab,ti OR 'heart atrial fibrillation':ab,ti OR 'heart atrium fibrillation':ab,ti OR 'heart fibrillation atrium':ab,ti OR 'non-valvular atrial fibrillation':ab,ti OR 'nonvalvular atrial fibrillation':ab,ti OR 'atrial fibrillation':ab,ti OR 'heart failure with preserved ejection fraction':ab,ti OR 'hfpef':ab,ti |
| #3 | 'catheter ablation'/exp |
| #4 | 'ablation, catheter':ab,ti OR 'catheter ablation':ab,ti |
| #5 | 'heart failure'/exp |
| #6 | 'backward failure, heart':ab,ti OR 'cardiac backward failure':ab,ti OR 'cardiac decompensation':ab,ti OR 'cardiac failure':ab,ti OR 'cardiac incompetence':ab,ti OR 'cardiac insufficiency':ab,ti OR 'cardiac stand still':ab,ti OR 'cardial decompensation':ab,ti OR 'cardial insufficiency':ab,ti OR 'chronic heart failure':ab,ti OR 'chronic heart insufficiency':ab,ti OR 'decompensatio cordis':ab,ti OR 'decompensation, heart':ab,ti OR 'heart backward failure':ab,ti OR 'heart decompensation':ab,ti OR 'heart incompetence':ab,ti OR 'heart insufficiency':ab,ti OR 'insufficientia cardis':ab,ti OR 'myocardial failure':ab,ti OR 'myocardial insufficiency':ab,ti OR 'heart failure':ab,ti OR 'heart failure with preserved ejection fraction':ab,ti OR 'hfpef':ab,ti |
| #7 | (((randomized AND controlled AND trial OR randomized) AND controlled AND trial OR rct OR clinical) AND trial OR cohort OR 'case control' OR clinical) AND study |
| #8 | #1 OR #2 |
| #9 | #3 OR #4 |
| #10 | #5 OR #6 |
| #11 | #7 AND #8 AND #9 AND #10 |
| #12 | #7 AND #8 AND #9 AND #10 AND [01-01-1966]/sd NOT [19-12-2023]/sd |

**4.Web of science**

| Search number | Query |
| --- | --- |
| #1 | TS=(Atrial fibrillation (Topic) OR Atrial Fibrillations (Topic) OR Fibrillation, Atrial (Topic) OR Fibrillations, Atrial (Topic) OR Auricular Fibrillation (Topic) OR Auricular Fibrillations (Topic) OR Fibrillation, Auricular (Topic) OR Fibrillations, Auricular (Topic) OR Persistent Atrial Fibrillation (Topic) OR Atrial Fibrillation, PersistentAtrial Fibrillation, Persistent (Topic) OR Atrial Fibrillations, Persistent (Topic) OR Fibrillation, Persistent Atrial (Topic) OR Fibrillations, Persistent Atrial (Topic) OR Persistent Atrial Fibrillations (Topic) OR Familial Atrial Fibrillation (Topic) OR Atrial Fibrillation, Familial (Topic) OR Atrial Fibrillations, Familial (Topic) OR Familial Atrial Fibrillations (Topic) OR Fibrillation, Familial Atrial (Topic) OR Fibrillations, Familial Atrial (Topic) OR Paroxysmal Atrial Fibrillation (Topic) OR Atrial Fibrillation, Paroxysmal (Topic) OR Atrial Fibrillations, Paroxysmal (Topic) OR Fibrillation, Paroxysmal Atrial (Topic) OR Fibrillations, Paroxysmal Atrial (Topic) OR Paroxysmal Atrial Fibrillations (Topic) ) and Preprint Citation Index (Exclude – Database) |
| #2 | TS=(Heart Failure (Topic) OR Cardiac Failure (Topic) OR Heart Decompensation (Topic) OR Decompensation, Heart (Topic) OR Heart Failure, Right-Sided (Topic) OR Heart Failure, Right Sided (Topic) OR Right-Sided Heart Failure (Topic) OR Right Sided Heart Failure (Topic) OR Myocardial Failure (Topic) OR Congestive Heart Failure (Topic) OR Heart Failure, Congestive (Topic) OR Heart Failure, Left-Sided (Topic) OR Heart Failure, Left Sided (Topic) OR Left-Sided Heart Failure (Topic) OR Left Sided Heart Failure (Topic) OR heart failure with preserved ejection fraction (Topic) OR HFpEF (Topic) OR Heart Failure, Diastolic (Topic) OR Diastolic Heart Failures (Topic) OR Heart Failure, Preserved Ejection Fraction (Topic) OR Heart Failure, Normal Ejection Fraction (Topic) OR Diastolic Heart Failure (Topic) ) and Preprint Citation Index (Exclude – Database) |
| #3 | TS=(Catheter ablation (Topic) OR Ablation, Catheter (Topic) OR Catheter Ablation, Transvenous (Topic) OR Transvenous Catheter Ablation (Topic) OR Ablation, Transvenous Catheter (Topic) OR Catheter Ablation, Electric (Topic) OR Electrical Catheter Ablation (Topic) OR Catheter Ablation, Electrical (Topic) OR Ablation, Electrical Catheter (Topic) OR Electric Catheter Ablation (Topic) OR Ablation, Electric Catheter (Topic) OR Ablation, Transvenous Electric (Topic) OR Electric Ablation, Transvenous (Topic) OR Transvenous Electric Ablation (Topic) OR Ablation, Transvenous Electrical (Topic) OR Electrical Ablation, Transvenous (Topic) OR Transvenous Electrical Ablation (Topic) OR Catheter Ablation, Radiofrequency (Topic) OR Radiofrequency Catheter Ablation (Topic) OR Ablation, Radiofrequency Catheter (Topic) OR Catheter Ablation, Percutaneous (Topic) OR Percutaneous Catheter Ablation (Topic) OR Ablation, Percutaneous Catheter (Topic) ) and Preprint Citation Index (Exclude – Database) |
| #4 | TS=(randomized controlled trial OR randomized controlled trial OR RCT OR clinical trial OR cohort OR case-control OR clinical study) and Preprint Citation Index (Exclude – Database) |
| #5 | #4 AND #3 AND #2 AND #1 and Preprint Citation Index (Exclude – Database) |

**5.Scopus**

| Search number | Query |
| --- | --- |
| #1 | ( TITLE-ABS-KEY ( "Atrial fibrillation" OR "Atrial Fibrillations" OR "Fibrillation, Atrial" OR "Fibrillations, Atrial" OR "Auricular Fibrillation" OR "Auricular Fibrillations" OR "Fibrillation, Auricular" OR "Fibrillations, Auricular" OR "Persistent Atrial Fibrillation" OR "Atrial Fibrillation, Persistent" OR "Atrial Fibrillations, Persistent" OR "Fibrillation, Persistent Atrial" OR "Fibrillations, Persistent Atrial" OR "Persistent Atrial Fibrillations" OR "Familial Atrial Fibrillation" OR "Atrial Fibrillation, Familial" OR "Atrial Fibrillations, Familial" OR "Familial Atrial Fibrillations" OR "Fibrillation, Familial Atrial" OR "Fibrillations, Familial Atrial" OR "Paroxysmal Atrial Fibrillation" OR "Atrial Fibrillation, Paroxysmal" OR "Atrial Fibrillations, Paroxysmal" OR "Fibrillation, Paroxysmal Atrial" OR "Fibrillations, Paroxysmal Atrial" OR "Paroxysmal Atrial Fibrillations" ) ) |
| #2 | ( TITLE-ABS-KEY ( "heart failure" OR "Cardiac Failure" OR "Heart Decompensation" OR "Decompensation, Heart" OR "Heart Failure, Right-Sided" OR "Heart Failure, Right Sided" OR "Right-Sided Heart Failure" OR "Right Sided Heart Failure" OR "Myocardial Failure" OR "Congestive Heart Failure" OR "Heart Failure, Congestive" OR "Heart Failure, Left-Sided" OR "Heart Failure, Left Sided" OR "Left-Sided Heart Failure" OR "Left Sided Heart Failure" OR "heart failure with preserved ejection fraction" OR "HFpEF" OR "Heart Failure, Diastolic" OR "Diastolic Heart Failures" OR "Heart Failure, Preserved Ejection Fraction" OR "Heart Failure, Normal Ejection Fraction" OR "Diastolic Heart Failure" ) ) |
| #3 | ( TITLE-ABS-KEY ( "Catheter ablation" OR "Ablation, Catheter" OR "Catheter Ablation, Transvenous" OR "Transvenous Catheter Ablation" OR "Ablation, Transvenous Catheter" OR "Catheter Ablation, Electric" OR "Electrical Catheter Ablation" OR "Catheter Ablation, Electrical" OR "Ablation, Electrical Catheter" OR "Electric Catheter Ablation" OR "Ablation, Electric Catheter" OR "Ablation, Transvenous Electric" OR "Electric Ablation, Transvenous" OR "Transvenous Electric Ablation" OR "Ablation, Transvenous Electrical" OR "Electrical Ablation, Transvenous" OR "Transvenous Electrical Ablation" OR "Catheter Ablation, Radiofrequency" OR "Radiofrequency Catheter Ablation" OR "Ablation, Radiofrequency Catheter" OR "Catheter Ablation, Percutaneous" OR "Percutaneous Catheter Ablation" OR "Ablation, Percutaneous Catheter" ) ) |
| #4 | ( ALL randomized AND controlled AND trial OR randomized AND controlled AND trial OR rct OR clinical AND trial OR cohort OR case-control OR clinical AND study ) ) |
| #5 | #1 AND #2 AND #3 AND #4 |

| Table S2A. CA versus medical therapy for patients with HFpEF. Newcastle-Ottawa Scale of bias assessment for non-randomized studies. | | | | | | | | | |
| --- | --- | --- | --- | --- | --- | --- | --- | --- | --- |
| Study | Selection | | | | Comparability | | Outcome | | |
|  | Representative-ness | Selection of  non-exposed | Ascertainment  of exposure | Outcome not present at start | Comparability on most important factors | Comparability on other risk factors | Assessment of outcome | Long enough follow-up (median≥1year) | Adequacy  (completeness) of follow-up |
| Arora et al2020 | * | * | * | * | * | * | * | - | * |
| Fukui et al 2020 | * | * | * | * | * | - | * | * | - |
| Rattka et al 2021 | * | * | * | * | * | - | * | * | * |
| *indicates criterion met; - indicates significant of criterion not met. | | | | | | | | | |

| TableS2B. CA for patients with HFpEF versus HFrEF. Newcastle-Ottawa Scale of bias assessment for non-randomized studies. | | | | | | | | | |
| --- | --- | --- | --- | --- | --- | --- | --- | --- | --- |
| Study | Selection | | | | Comparability | | Outcome | | |
|  | Representative-ness | Selection of  non-exposed | Ascertainment  of exposure | Outcome not present at start | Comparability on most important factors | Comparability on other risk factors | Assessment of outcome | Long enough follow-up (median≥1 year) | Adequacy  (completeness) of follow-up |
| Aldaas et al 2020 | * | * | * | * | * | - | * | * | - |
| Black-Maier et al 2017 | * | * | * | * | * | - | * | * | - |
| Cha et al 2011 | * | * | * | * | * | - | * | * | * |
| Eitel et al 2019 | * | * | * | * | * | - | * | * | * |
| Fujimoto et al 2022 | * | * | * | * | * | - | * | * | * |
| Ichijo et al 2018 | * | * | * | * | * | - | * | * | - |
| Ishiguchi et al 2022 | * | * | * | * | * | - | * | * | * |
| Qiao et al 2022 | * | * | * | * | * | - | * | * | * |
| Chen et al 2023 | * | * | * | * | * | - | * | - | * |
| *indicates criterion met; - indicates significant of criterion not met. | | | | | | | | | |

| Table S2C. CA for patients with HFpEF versus without HF. Newcastle-Ottawa Scale of bias assessment for non-randomized studies. | | | | | | | | | |
| --- | --- | --- | --- | --- | --- | --- | --- | --- | --- |
| Study | Selection | | | | Comparability | | Outcome | | |
|  | Representative-ness | Selection of  non-exposed | Ascertainment  of exposure | Outcome not present at start | Comparability on most important factors | Comparability on other risk factors | Assessment of outcome | Long enough follow-up (median≥1 year) | Adequacy  (completeness) of follow-up |
| Aldaas et al 2020 | * | * | * | * | * | - | * | * | - |
| Cha et al 2011 | * | * | * | * | * | - | * | * | * |
| Zylla et al 2022 | * | * | * | * | * | - | * | * | * |
| Rattka et al 2020 | * | * | * | * | * | - | * | * | * |
| Yamauchi et al 2020 | * | * | * | * | * | - | * | * | * |
| Chen et al 2023 | * | * | * | * | * | - | * | - | * |
| *indicates criterion met; - indicates significant of criterion not met. | | | | | | | | | |

FigureS1. Cochrane risk of bias assessment for randomized studies. Cochrane risk of bias assessment. A) Cochrane Risk of Bias Graph. B) Cochrane Risk of Bias Summary.


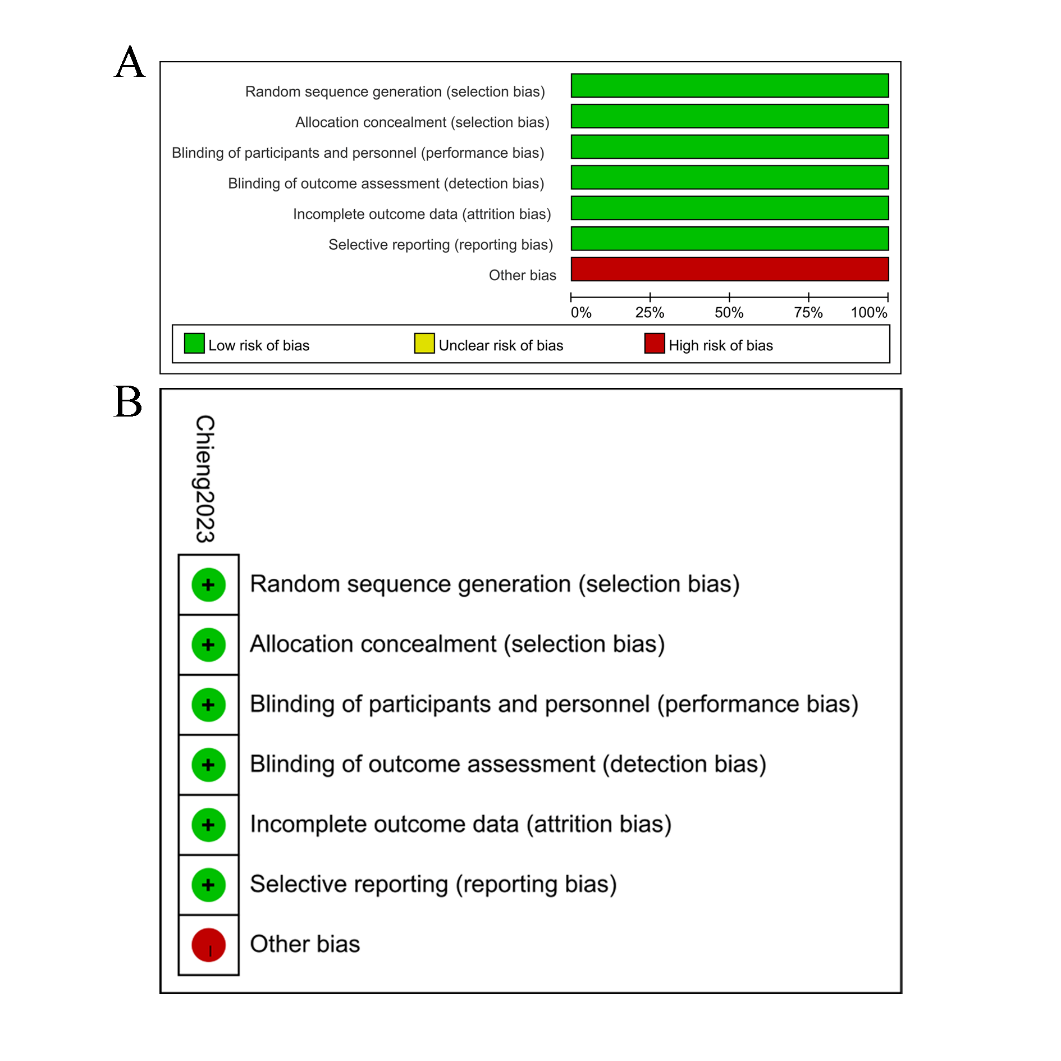

Supplement: Supplementary file 1 [file Datasheet1.docx]
